# MIMAS: an innovative tool for network-based high density oligonucleotide microarray data management and annotation

**DOI:** 10.1186/1471-2105-7-190

**Published:** 2006-04-05

**Authors:** Leandro Hermida, Olivier Schaad, Philippe Demougin, Patrick Descombes, Michael Primig

**Affiliations:** 1 Biozentrum and Swiss Institute of Bioinformatics Klingelbergstrasse 50-70 CH-4056 Basel Switzerland; 2 Genomics Platform, NCCR Frontiers in Genetics, Geneva University Medical Center 1, Rue Michel-Servet CH-1211 Geneva Switzerland

## Abstract

**Background:**

The high-density oligonucleotide microarray (GeneChip) is an important tool for molecular biological research aiming at large-scale detection of small nucleotide polymorphisms in DNA and genome-wide analysis of mRNA concentrations. Local array data management solutions are instrumental for efficient processing of the results and for subsequent uploading of data and annotations to a global certified data repository at the EBI (ArrayExpress) or the NCBI (GeneOmnibus).

**Description:**

To facilitate and accelerate annotation of high-throughput expression profiling experiments, the Microarray Information Management and Annotation System (MIMAS) was developed. The system is fully compliant with the Minimal Information About a Microarray Experiment (MIAME) convention. MIMAS provides life scientists with a highly flexible and focused GeneChip data storage and annotation platform essential for subsequent analysis and interpretation of experimental results with clustering and mining tools. The system software can be downloaded for academic use upon request.

**Conclusion:**

MIMAS implements a novel concept for nation-wide GeneChip data management whereby a network of facilities is centered on one data node directly connected to the European certified public microarray data repository located at the EBI. The solution proposed may serve as a prototype approach to array data management between research institutes organized in a consortium.

## Background

Microarray-based approaches have become an important tool to help better understand fundamental biological processes like the mitotic cell cycle [1], meiotic development [2,3], tissue-specific gene expression [4], or the etiology and progression of diseases [5,6]. Large-scale transcriptional profiling is still the most common application of microarrays [7-9] although other approaches such as analysis of DNA polymorphisms [10], DNA copy number changes [11] and protein-DNA interactions [12] are becoming increasingly important (for a comprehensive review, see [13]).

There is a range of microarray platforms available today, each utilizing different technologies for manufacture, detection and labeling. The most common platforms are either based on adhesion of DNA molecules applied by a robotic device (spotted arrays) or *in situ *synthesis of covalently attached short oligonucleotide probes onto a glass support (GeneChips). A major issue is that cross-platform compatibility tends to be poor since often only a small subset of genes detected as differentially expressed are found with both array types [2,9,14]. Although it was thought that manufacturing arrays in-house would be an affordable solution issues such as batch-to-batch reproducibility and poor data quality have emerged, making commercial array systems a desirable option. Such platforms also have the advantage that they include standard protocols and reaction kits for each step of the preparation and hybridization process. Moreover, experiments carried out with GeneChips are known to yield robust and highly reproducible data across research facilities and even across species [3]. The classical architecture of GeneChips is based on synthesizing a set of oligonucleotide probes complementary to mRNAs encoded by putative genes previously defined during an error-prone process called genome annotation [15]. A new type of array covers the whole genome sequence of the target organism by overlapping ("tiling") oligonucleotides and therefore should detect all transcripts that are encoded by the genome. Preliminary results indicate that a very large number of transcripts failed to be detected in the predominantly protein-oriented annotation efforts carried out so far [16,17].

Because producing microarray data is still expensive (and will remain so for the foreseeable future), a major goal of any repository is to maximize the usefulness and value of the data by providing a framework for sharing and "recycling" data into repeated rounds of analysis. To meet this challenge, coordination of the array data production process carried out at different microarray facilities equipped with similar array systems is essential [14,18]. It is furthermore crucial that comprehensive information be gathered about each step in the data production process and this information be described in a standardized way. Metadata (information about data) is vital for data analysis and comparison and obtaining that information represents one of the major hurdles facing microarray facilities today. The Microarray Gene Expression Data Society (MGED, [19]) was founded to ensure that microarray experiments are performed in a scientifically sound manner and to establish standards for microarray data annotation, storage and exchange [20]. MGED's major standardization projects are the Minimum Information About a Microarray Experiment (MIAME) guidelines [21], the MicroArray and Gene Expression (MAGE) data representation and exchange standard [22], and the MGED Ontology for microarray experiment and biological sample annotation [23]. Annotating microarray data according to MIAME guidelines has now become mandatory for publishing in most scientific journals [24]. Currently there are certified repositories in Europe (EBI, ArrayExpress), in the US (NCBI, GeneOmnibus), and in Japan (DDBJ, CIBEX) which hold published data for all types of microarray platforms [25-28].

Several open source and commercial solutions have been developed to store and annotate microarray data [29-33]. However, they evolved from the need to support custom two-color spotted arrays whose data and description require a much more generalized and complicated system than data from GeneChips. In addition, the graphical user interface (GUI) of these solutions cannot be as straightforward or intuitive as one tailored for one array type such as GeneChips. Such a focused approach was implemented for example in a recently reported standalone application for array data storage and annotation [34]. Here we report the development of a novel and focused solution for consortium-wide and standardized GeneChip data management in conjunction with the EBI's ArrayExpress repository [35]. The web-accessible Microarray Information Management and Annotation System (MIMAS) is based upon a sophisticated GUI and a scalable relational database. It is designed to hold manually annotated expression data from several research facilities organized within a consortium. MIMAS, which may serve as a prototype for GeneChip communities in other consortia or countries, is freely available upon request for academic institutes under the GNU license [36].

## Construction and content

### The database model

The database model, shown in Additional File 1, is designed to efficiently and flexibly fulfill the design requirements of MIMAS. A major goal of the project was to keep data storage as straightforward and manageable as possible while retaining the capability to grow and evolve. The database model can be divided into five functional areas: *Controlled Vocabulary & Array Library*, *Data Repository*, *User/Group Security & Management*, *Experiment Upload/Working*, and *Web Management*.

The *Controlled Vocabulary & Array Library *contains the resource and display information used to describe and annotate experiments. The main *Attribute *table contains information on annotation fields used to describe a microarray experiment such as "*Author(s)*", "*Organ/Organism Part*", "*Cell/Tissue Separation Technique*" or "*Image Analysis Algorithm*". The *Attribute *table includes the annotation fields described in the MIAME guidelines and the MGED Ontology as they apply to GeneChips. Attributes are grouped into their respective MIAME or other functional categories in the *Attr_group *table. These categories include array design, experiment description and design, biomaterial properties, hybridization protocols, image analysis, data processing, and experimental factor details. *Attribute *fields which require controlled vocabularies are maintained in the *Attr_detail *table. Depending on the nature of the *Attribute *field, entries in the *Attr_detail *table can be terms used to describe the field or allowed units if the field requires numerical input and a unit. The latest MGED Ontology is populated in the *Attr_detail *table when it is available for the respective *Attribute *field. The *Attr_detail *table also holds unit conversion information for controlled vocabulary unit groups. Unit conversion information is used to provide powerful querying of the MIMAS repository. Since controlled vocabulary lists for a particular *Attribute *can grow to be very long, *Attr_detail *entries can be grouped into display categories in the *Attr_detail_group *table. Controlled vocabulary grouping facilitates location of the appropriate controlled vocabulary term in a long list in the web application. The *Attribute*, *Attr_group*, *Attr_detail*, and *Attr_detail_group *tables are designed for flexible growth and new fields, groups and vocabularies can be easily added when needed as well as deprecated when they become obsolete. These tables also maintain all the display information to dynamically generate and control the format of many pages in the MIMAS web application. Annotation field groupings, display orders, default selections, controlled vocabulary structures, popup descriptions, and input field types are stored in these tables and allow for fast and flexible reconfiguration of the web application pages. The *Array_series *and *Array *tables contain the necessary descriptive information for GeneChips. This information includes official and alternate array names, number of features, and array type. The *Array_series *and *Array *tables are populated by the GeneChip CDF loader script which uses CDF files as input.

The *Data Repository *area of the database stores the transformed annotations and data for completed microarray experiments and is used as the basis for querying MIMAS. Fast and detailed queries into the repository can be executed due to the straightforward, denormalized structure of the schema. The *Data_file *table stores the compressed data file contents for the raw probe-level CEL and derived probeset (gene)-level files (such as GCOS/MAS TXT, RMA, GC-RMA). Meta information about the data files such as the unique file fingerprint, file type version and source array is also stored in the table. File fingerprinting via an md5 checksum is used to avoid data redundancy that might occur when users accidentally upload identical data files under two different names. The *Data_file *table is capable of storing any data file type, thus allowing for incorporation of future data file formats. The main *Sample *table holds the unique identifier for each repository sample and the link to the sample owner in the *User *table and data files in the *Data_file *table. Each *Sample *is fully described and annotated in the *Sample_attribute *table. *Sample_attribute *entries reference an *Attribute *field and are stored as a character string (free text), numeric value, or link to a controlled vocabulary term in the *Attr_detail *table. If the *Sample_Attribute *is a numeric value then it could also contain a link to a controlled vocabulary unit in the *Attr_detail *table if applicable.

The *User/Group Security & Management *area stores detailed information about users, groups, laboratories and organizations as well as the *Sample *access privileges of users and groups. The *User*, *Lab *and *Organization *tables contain the respective information about these entities. Users belong to a laboratory which itself belongs to an organization. Users can also belong to various groups in the *Group *table through relationships defined in the *User_to_group *table. User and group read and write privileges to repository samples are controlled in the *User_privilege *and *Group_privilege *tables.

The *Experiment Upload/Working *area of the database holds microarray experiments and supplementary information for uploads via the MIMAS web application. The main *Up_experiment *table holds experiment entries, their owner and possible curator and the state of each experiment. Experiment samples and information about their associated uploaded files are kept in the *Up_sample *and *Up_sample_file *tables. *Up_sample *entries are grouped into experimental conditions which are stored in the *Up_exp_condition *table. Experiment-level annotations for each *Up_experiment *are stored in the *Up_exp_attribute *table and experimental condition- and sample-level annotations are stored in the *Up_sample_attribute *table.

The *Web Management *area in the database model stores information associated with web sessions, job requests and user information alerts. User job requests, such as requests to download samples and annotations from the repository, are stored in the *Job *table. Alerts and other important information generated by system services and displayed to users are stored in the *Alert *table and MIMAS web application login session data are stored in the *Session *table.

### The web application

The MIMAS web application is a browser-based GUI used to upload, annotate and manage microarray experiments as well as search for and download these data (Additional Files 2 and 3). A major goal during its development was to provide scientists with an intuitive, uncomplicated and streamlined tool for these tasks. In addition, a key requirement of the project was to provide a client application easily available to the GeneChip facilities located across Switzerland. Choosing the ubiquitous web browser as the client application was obvious because of the advantages over other client systems with regards to compatibility, user familiarity and application updates.

The external pages of the MIMAS web site provide general information, links to resources, user registration and login capabilities. Registered users can log in to MIMAS and obtain access to the tools and their personal workspace. Alerts, completed job requests and other user information are displayed on the user home page of the personal workspace. Internal navigation is performed using the main navigation area located on the left side of the browser window. At the top of the navigation area are links to the main internal menus. These links represent the four major internal areas: User Information and Management "*User Home*", Microarray Data Analysis "*Analysis Toolkit*", Microarray Experiment Uploads and Annotation "*Experiment Uploads*", and Microarray Data Search and Retrieval "*Search Repository*". Each main menu contains a corresponding detail menu with additional links.

### Experiment upload and management

Users manage and create microarray experiments through the *Experiment Uploads *navigation menu. The experiment upload management page allows for the creation of new experiments, displays the status of experiments already stored, and provides links to edit or remove ongoing experiments. Regardless of their state of completion, MIMAS maintains all experiments in the user's workspace indefinitely unless the user chooses to remove them. Through the central management page multiple experiments can be accessed and processed simultaneously. First, GeneChip raw data CEL and corresponding derived data files are uploaded via the *File Upload *page. Then, experimental conditions are created and the GeneChip series is selected in the *Sample Relationships *page and samples created in the *File Upload *page are mapped to their appropriate experimental condition and microarray. Sample mapping data are used to automatically determine quality control and replicate information relevant for the experiment. Detailed MIAME and additional MIMAS annotation is completed on the *Experiment Information *and *Sample Attributes *pages. On the *Experiment Information *page, users complete annotations which apply to all the samples in their experiment, such as "*Author(s)*", "*Experiment Design Type*" and "*Experimental Factors*". On the *Sample Attributes *pages, annotations for individual samples or for all the samples in an experimental condition are completed, such as "*Sex/Mating Type*", "*Disease State*" and "*Growth Conditions*". On both the *Experiment Information *and *Sample Attributes *web pages, fields are grouped into their respective MIAME categories: *Organization Information and Contacts*, *Experiment Design Information*, *Microarray Technology and Quality Control, Biomaterial Characteristics*, *Hybridization Protocol*, *Image Analysis and Data Processing*, and *Experimental Factor Details*.

On the *Experiment Information *and *Sample Attributes *pages, annotation fields have different input interfaces depending on what type of field they are and what kind of control is placed on them (Additional File 2, panel A). MIMAS presently has five field types: controlled vocabulary fields allowing a single selection presented as drop-down menus, controlled vocabulary fields allowing multiple selection presented as add/remove menus, free text fields allowing short phrase, term, identifier or name input presented as single line text boxes, free text fields allowing free-form paragraph and descriptive input presented as multi-line text boxes, and numeric fields presented as numeric input boxes with controlled vocabulary unit drop-down menus where appropriate. To facilitate the laborious process of filling in all of the sample attributes for each sample, MIMAS provides two features on the *Sample Attributes *page: the ability to fill in sample attributes once for all the samples in an experimental condition and the ability to copy sample attributes from any sample to any other sample in the experiment. These tools make it extremely easy to complete the frequently recurring sample attributes in a microarray experiment. Controlled vocabulary fields obtain terms from the MIMAS controlled vocabulary library which is populated with the latest available MGED Ontology [37]. Since few controlled vocabularies have been fully developed and the MGED Ontology is at present limited, MIAME stresses that annotation tools should still attempt to use controlled vocabularies and provide ways of building their own controlled vocabulary lists. MIMAS provides this important capability by allowing the creation of user-defined controlled vocabulary terms during experiment annotation (panel B). During experiment curation these user-defined terms are checked for their accuracy and validity and, upon approval, are added to the controlled vocabulary library. After the completion of all of the *Sample Attributes *pages, an *Upload Summary *page is presented before the experiment is submitted for curation. After submitting the experiment to curation, the user is brought back to the experiment management page where they can see the experiment is now in the curation process. The experiment cannot be edited by the user at this point.

The GUI and web application contain built-in logic to support accurate experiment annotation and data file upload. For example, on the *File Upload *page uploaded files are parsed and checked in detail to make certain their contents are authentic and match the CDF information held in the MIMAS *Array Library*. In addition, raw data CEL files must be properly paired to their derived data files. On the *Sample Relationships *page replicate numbers must match between experimental conditions or the user is warned to override or correct the mistake. On the *Experiment Information *and *Sample Attributes *pages there are required fields that users must complete to submit their experiment and if certain linked annotation fields contradict each other an error message is issued to the user telling them that their field entries do not make sense. Finally, built-in logic and error verification is supplemented by the manual curation process.

### Sample search and retrieval

MIMAS users can search for and download samples that they own or have access to view through the *Search Repository *main menu navigation link (Additional File 3). The search tool has the capability of performing queries ranging from very simple or broad (e.g. obtaining samples from a single experiment or owner) to complex and focused (e.g. obtaining treated samples which used a specific treatment delivery method or sample hybridizations which used a particular cDNA synthesis kit). This enables life scientists to retrieve datasets that have a biological focus (e.g. experiments which study gene expression in germ cells or liver) while data analysts can find samples with specific characteristics across many different experiments (e.g. all non-treated samples or samples treated with a specific compound).

MIMAS repository searching is performed in two steps. First, major search criteria are selected from all annotation fields used to describe microarray experiments. The major criteria selection menu has the annotation fields intuitively grouped into their respective MIAME categories and in the same order as they are found in the experiment upload annotation pages. Second, the detailed query is prepared using the individual interfaces for each major search criterion. MIMAS currently has four types of query interfaces depending on the type of annotation field selected as a criterion: controlled vocabulary fields are searched using a multiple selection add/remove menu with the controlled vocabulary list for that field, free text fields are searched using a string search box with string search modifiers, numeric fields are searched using a numeric search box with numeric search modifiers and, if the field has units, a single selection drop down menu with the controlled vocabulary units list, and date fields are searched using a set of date range boxes. The sample search results page(s) lists those repository samples found which meet the search criteria and summary information for each sample. Users can click to view the complete details of each sample in the result list and they can also choose from the list which samples they would like to download. Download requests are processed in the background by the MIMAS job service and when they are complete, a link to the requested data files and annotations is provided on the personal user home page.

### Experiment curation

The MIMAS curation tool is presently a command line application used by curators to view annotation summaries of submitted experiments and to curate in detail any user-defined controlled vocabulary terms. Curators can approve or send an experiment back to the user to make any changes, corrections or clarification. Users are notified through their personal user home page and via email when a submitted experiment has been sent back for revision and for what reasons. The returned experiment can then be edited by the user and they can resubmit it once they have completed any requested changes. When a submitted experiment is approved by a curator, it is automatically signaled to be transformed into the MIMAS *Data Repository *and made available for searching and data retrieval by the transformation service. Users are also notified through their personal user home page and via email when an experiment has been approved. They will also see in their experiment upload management page that the experiment is in the repository. Completed experiments in the repository can be updated at a later time by contacting the MIMAS administrator to bring the experiment back into an editable state.

At this point in the submission process any other services or tools can be plugged into MIMAS to make data and annotation available to external databases or applications. Presently, one major service is implemented which uses the MIMAS SOAP and database APIs. When a user's experiment is completed, curated and then made available to the repository, the MIMAS-GeneSpring connector service transforms the data and annotations and then publishes it to a commercial system called GeneSpring Workgroup. This product provides users a centralized, server-based workspace to do their analyses with the corresponding GeneSpring GX client application.

## Utility

Important biological problems, such as growth, development and diseases that have been studied using genetics, biochemistry and molecular biology over the past decades are now also investigated using genome biological approaches. However, high-throughput experiments yield enormous amounts of quantitative data that require an extensive computational infrastructure for storage, analysis and interpretation. The key point is that the current generation of life scientists is not necessarily acquainted with large-scale computing installations; neither do they in general possess the extensive programming skills required to implement complex mathematical concepts for genome-wide data analysis. In order to fully exploit the potential of the emerging discipline of molecular systems biology, it is clear that molecular biologists and biomedical researchers will need appropriate training, comprehensive bioinformatics tools and protocols for the production of reliable data [38,39].

High-density oligonucleotide (GeneChip) microarray experiments are based on a robust method but certain parameters remain hard to control such as particular features or settings of the equipment, specifics of the protocol and user-dependent aspects [14,18,40]. It is therefore crucial to standardize raw data production, processing, annotation and storage so as to maximize the value and sharing capacity of the data. We propose a network solution for GeneChip data management whereby one location in a given consortium (or a multi-national consortium of research institutes) provides a central facility hosting the data and system and enabling scientists to describe (annotate) them via the Internet according to the international MIAME convention. That system, at the request of the user, then automatically manages preparation and transfer of that data (using MAGE-ML) upon publication of the corresponding manuscript to at least one of the certified array data repositories at the EBI, the NCBI or DDBJ [25-28].

## Discussion

The accurate description of complex expression profiling (and DNA analysis) studies requires as much case as the experimental work itself. The MIAME guidelines are continually evolving and MIMAS has been built with this in mind. The system also allows users not only to exploit existing descriptors (annotation terms) but also to actively build, within a community effort, the controlled vocabulary needed for proper description of data. This capability is important since the structure and strategy of the MGED Ontology is being debated [41]. The future will show to what extent other ontologies (description of concepts and relationships for the purpose of knowledge sharing and reuse) used to annotate DNA sequences, gene products (GeneOntology, [42]) and cells [43] can be employed in array experiment annotation [44-46].

### Comparison with other solutions

Other solutions for array data management that were developed in recent years have either been abandoned (GeneX, [47]), or they are complex custom-tailored solutions (often designed predominantly for cDNA microarrays) not meant to be implemented by many different research facilities (Stanford Microarray Database, SMD [48]). MiMiR is a recently described GeneChip data storage and annotation solution [34]. However, as opposed to a web-browser based solution such as MIMAS, it is not optimized for remote access and application updates. These two features are essential for network-based usage by several research facilities organized within a consortium. GEO (NCBI) [25] and ArrayExpress (EBI) [35] are global repositories not suitable for local platforms that need secure access as well as flexible and specific annotation options not necessarily implemented in an archiving system that covers all currently available high-throughput platforms. Moreover, customization of comprehensive solutions is time-consuming and expensive and often, due to unstable code releases, not feasible. Finally, it should be mentioned that the EBI and NCBI do not (yet) have the capacity to store the rapidly growing amount of array data information and therefore they cannot be used by thousands of researchers for "private" data archiving. As a consequence it is crucial to develop flexible local management solutions that directly feed into the certified repositories thereby speeding up the tedious process of data annotation and uploading via the Internet.

### Future work

In the MIMAS system and database the capability is already available to provide descriptions of vocabulary terms so that when a user hovers over or clicks on a term in a web application menu the description is displayed in a popup window. We are in the process of completing these descriptions to make this useful feature fully functional. In the case of MGED Ontology, hyperlinks leading to the MGED web page will also be added, where the formal definition of the vocabulary term is given. As far as new user-defined terms are concerned, a detailed description of their meaning and source will be requested before they are added to the system. This pool of terms will be important for further development of a comprehensive array data ontology by MGED [49].

Our current plan is to develop the MIMAS analysis toolkit which will provide solutions for quality control, visualization and interpretation of microarray data in the repository. We first intend on integrating MIMAS with the *Remote Analysis Computation for Gene Expression Data *(RACE) suite of microarray data analysis tools [50]. This is a web application written in Perl and R and designed to pre-process and quality control GeneChip data using open source packages provided by the *BioConductor *project [39,51]. Moreover, users will be able to mine their expression data with *GoCluster*, an R tool that reveals significant enrichment of GeneOntology terms within clusters of co-expressed genes obtained with a variety of clustering algorithms [52]. Our ultimate aim is to develop an integrated and convenient suite of open source applications that are straightforward to use by life scientists who wish to carry out and fully comprehend their array work themselves rather than outsourcing it to companies or to pure service facilities.

## Conclusion

MIMAS is a MIAME-compliant relational database implemented in Oracle (and portable into MySQL) for high-density oligonucleotide microarray data archiving, annotation and export used at several GeneChip facilities in Switzerland. The system was developed for a large multi-facility user base and its most important features include (i) a modular and easily extendable database model, (ii) a large data storage capacity (we are currently uploading approximately 1500 hybridization experiments), (iii) a sophisticated submission interface that enables scientists to develop the controlled vocabulary used to describe array experiments, (iv) extensive and detailed search functions that allow retrieval of data according to numerous criteria including experimental conditions, biological features and sample types. MIMAS's flexible and scalable architecture will enable it to hold very large amounts of data to be expected from the upcoming generation of DNA analysis and tiling expression arrays. These novel GeneChips are likely to help gain unprecedented insight into the activation, regulation and ultimately the function of most, if not all protein and RNA gene products encoded in a genome.

## Availability and requirements

**Figure 1:**
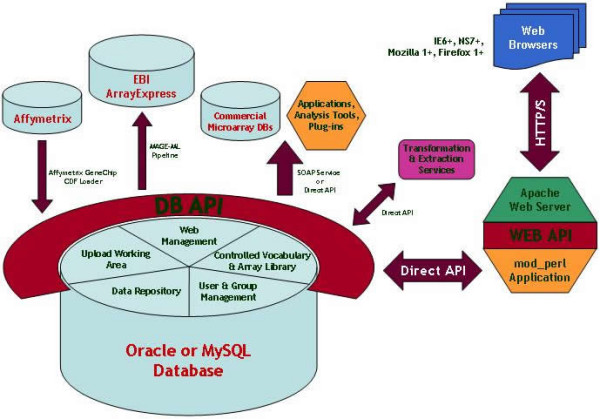
The MIMAS system architecture. A schematic overview of the database and its services as well as web front-end components is shown.

MIMAS release 1.0 was developed using open source and freely available software development tools. The system software and installation instructions are available for downloading at the system's Internet portal [36]. The system architecture consists of four major components, shown in Figure 1: *Database*, *Application Programmer Interface *(*API)*, *Web Application*, and *Auxiliary Services, Plug-ins & Loaders*.

The *Database *component houses information stored by the system in a relational database. MIMAS can run on the open source MySQL 4.1 [53] or, if a higher level of scalability is needed, commercial Oracle 9.2 [54] database management systems (DBMS). An easy-to-use master creation script is provided to automatically generate the MIMAS database schema and entities such as database users, data files, layouts and tablespaces. Depending on which DBMS is chosen to implement the MIMAS database component, construction of the underlying physical database structure is optimized utilizing available features of that particular DBMS. The script also populates the database with essential seed data and prompts the administrator for installation-specific information during its execution. The standalone, task-specific code modules used by the script can also be customized to meet local requirements.

The *API *component, a set of object-oriented classes and modules used to develop applications to interact with the system, is written in Perl and requires installation of Perl 5.8 or greater. The database part of the *API *provides a complete object-oriented interface to the MIMAS database via an object-relational mapping and storage management layer. The web part of the *API *is a set of classes and templates used to construct and display the components of the MIMAS GUI and web application.

The *Web Application *component is a mod_perl and JavaScript web application built using the Apache 1.3.33 web server [55] and mod_perl 1.29 [56]. MIMAS can run using the standard hypertext transfer protocol (HTTP) or SSL HTTPS protocol. The Apache configuration file used with MIMAS is provided in the distribution. The GUI runs on Internet Explorer 6.0+, Mozilla Firefox 1.0+ and Netscape Navigator 7.0+ using their integrated JavaScript interpreters.

The *Auxiliary Services, Plug-ins & Loaders *component is a set of Perl scripts and daemons which manage various tasks such as transformation of completed microarray experiments, job requests executed by users, and integration of data with external databases. In addition to the core Perl distribution, the MIMAS system and components require various Perl modules such as DBI, DBD::mysql, DBD::Oracle, Apache::DBI, FindBin, SOAP::Lite, MIME::Base64, MIME::Lite, and Archive::Zip. They are freely available for download from the Comprehensive Perl Archive Network (CPAN, [57]).

The recommended system configuration is to install the web server and web application on a dedicated server and the database component on a separate database server, which can be shared with other workloads. Services, plug-ins and data loaders can run from the web server if needed but can also run from other servers to distribute load. The system code was written to be portable and operating system/architecture independent. Since the software components required by MIMAS (MySQL, Oracle, Perl, Apache, mod_perl) are capable of running on many common operating systems (UNIX, Linux, Windows), the distribution can be installed on any one or a mixture of these. Once MIMAS is installed, configuration and customization to the web application and services are done through a central configuration file.

## Abbreviations

API (Application Programmer Interface), CPAN (Comprehensive Perl Archive Network), DBMS (Database Management System), GCOS (GeneChip Operating System), GUI (Graphical User Interface), HTTP (Hypertext Transfer Protocol), MAGE (MicroArray and Gene Expression), MAS (Microarray Analysis Suite), MGED (Microarray Gene Expression Data), MIAME (Minimal Information about a Microarray Experiment), RMA (Robust Multi-array Average), SOAP (Simple Object Access Protocol), SSL (Secure Sockets Layer).

## Authors' contributions

LH developed the system and drafted the manuscript. OS, PD, and PD contributed to the development of the database model and the graphical user interface. MP participated in project conceptualization and design and drafting of the manuscript. All authors read and approved the final manuscript.

## Supplementary Material

Additional File 1 The MIMAS database model. Database areas are color-coded. Yellow tables represent *Controlled Vocabulary & Array Library*, blue tables *Data Repository*, red tables *User/Group Security & Management*, green tables *Experiment Upload/Working*, and purple tables *Web Management*. Table relationships are indicated by appropriate symbols in standard ERD format. Click here for file

Additional File 2 The MIMAS Web GUI. Panel A shows the Experiment Design Annotation Page and panel B displays the Extensible Controlled Vocabulary System. Examples of descriptors provided by the system or added by the user are shown. Click here for file

Additional File 3 The MIMAS Web GUI – Repository Search Page. An example of possible search options is shown. Click here for file
