# Supplementary figures and images for: MIMAS: an innovative tool for network-based high density oligonucleotide microarray data management and annotation
Source: BMC Bioinformatics. 2006 Apr 5;7:190. doi: 10.1186/1471-2105-7-190 (PMC1459208; doi:10.1186/1471-2105-7-190)

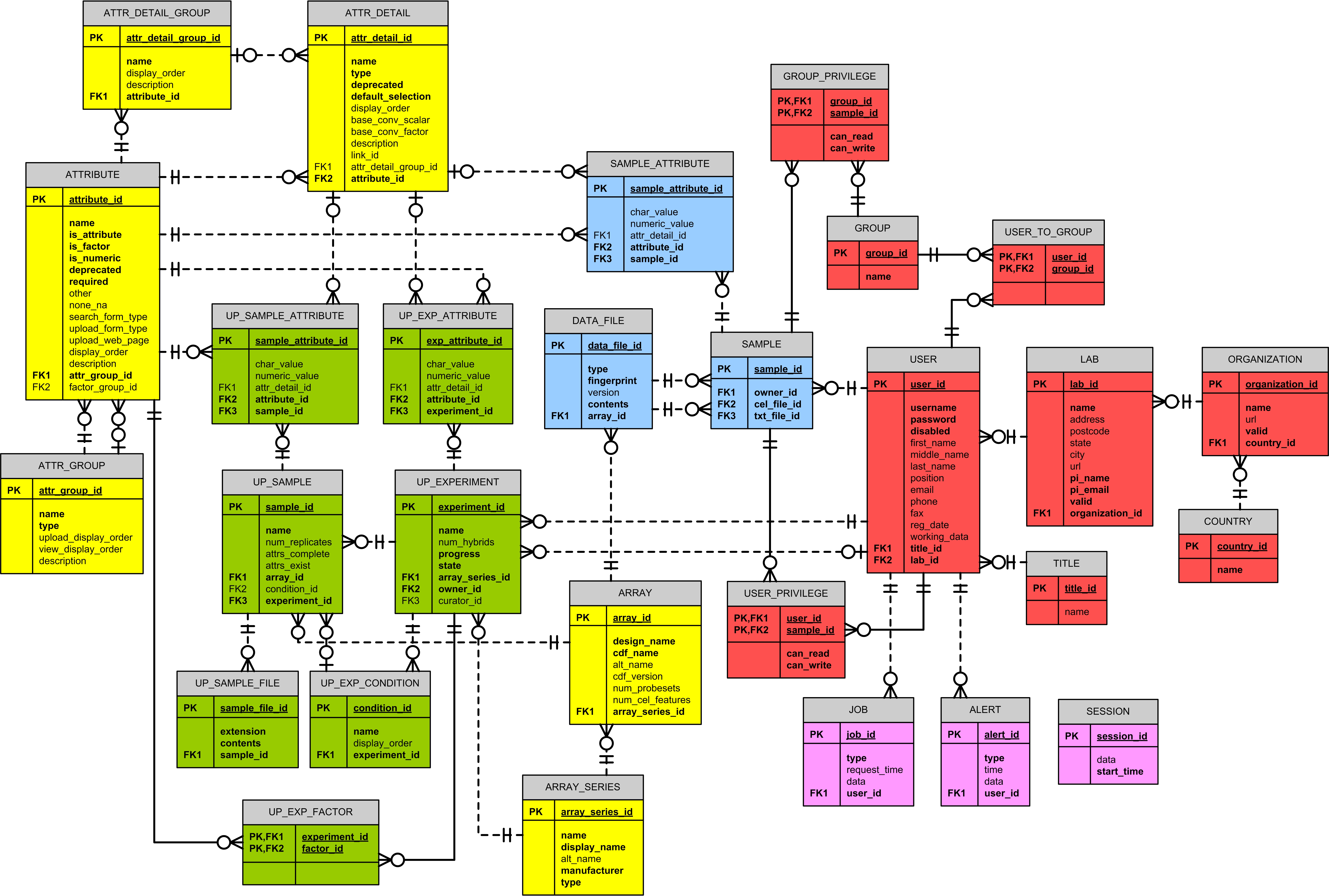

Supplement: Additional File 1 — The MIMAS database model. Database areas are color-coded. Yellow tables represent Controlled Vocabulary & Array Library, blue tables Data Repository, red tables User/Group Security & Management, green tables Experiment Upload/Working, and purple tables Web Management. Table relationships are indicated by appropriate symbols in standard ERD format. [file 1471-2105-7-190-S1.jpeg]

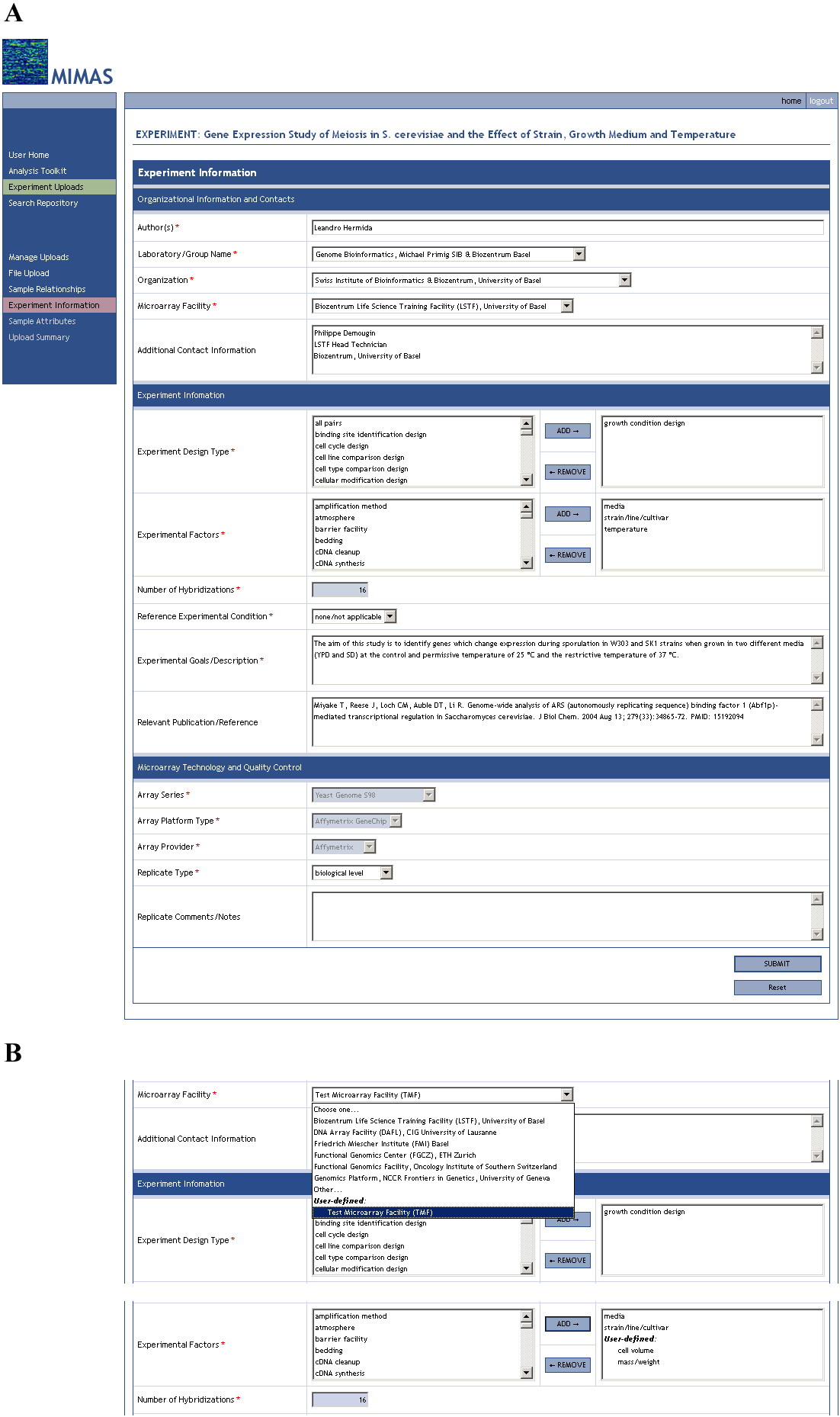

Supplement: Additional File 2 — The MIMAS Web GUI. Panel A shows the Experiment Design Annotation Page and panel B displays the Extensible Controlled Vocabulary System. Examples of descriptors provided by the system or added by the user are shown. [file 1471-2105-7-190-S2.jpeg]

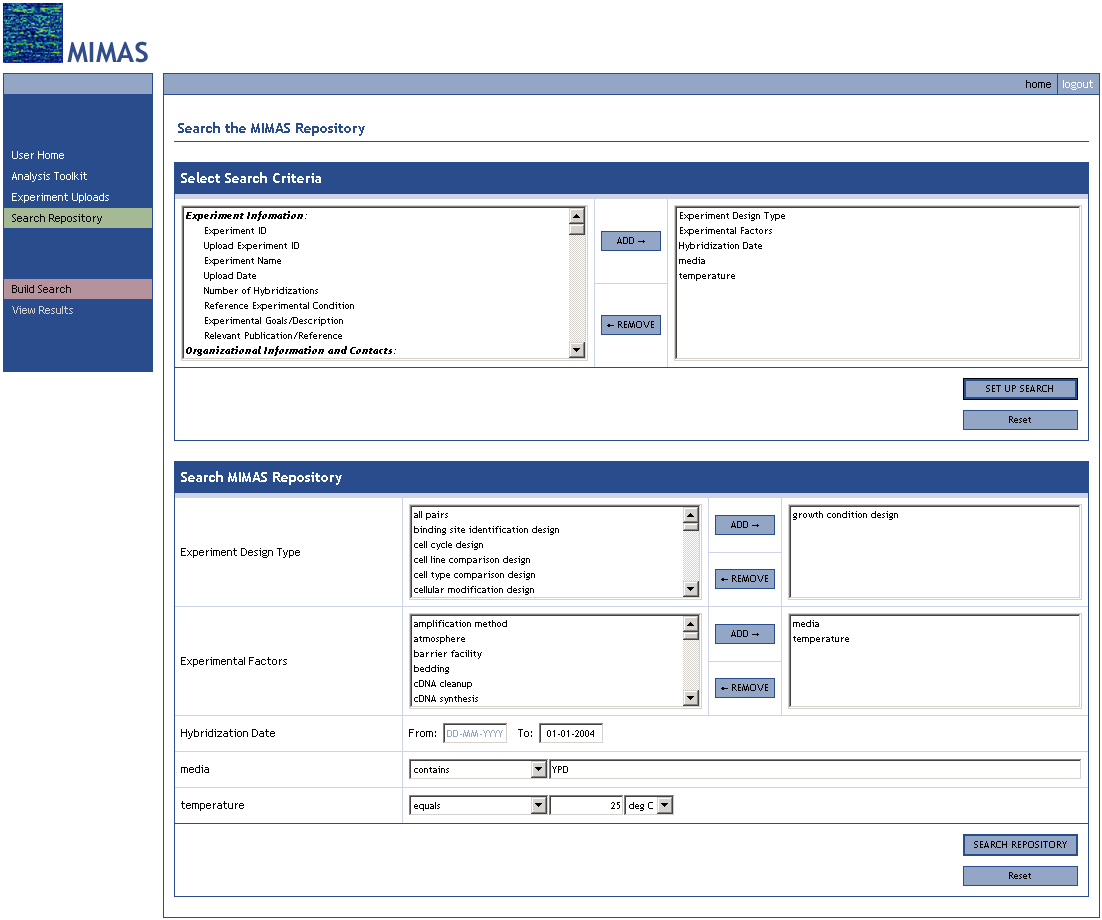

Supplement: Additional File 3 — The MIMAS Web GUI – Repository Search Page. An example of possible search options is shown. [file 1471-2105-7-190-S3.jpeg]
